# Usage of the Anemia Control Model Is Associated with Reduced Hospitalization Risk in Hemodialysis

**DOI:** 10.3390/biomedicines12102219

**Published:** 2024-09-28

**Authors:** Mario Garbelli, Maria Eva Baro Salvador, Abraham Rincon Bello, Diana Samaniego Toro, Francesco Bellocchio, Luca Fumagalli, Milena Chermisi, Christian Apel, Jovana Petrovic, Dana Kendzia, Jasmine Ion Titapiccolo, Julianna Yeung, Carlo Barbieri, Flavio Mari, Len Usvyat, John Larkin, Stefano Stuard, Luca Neri

**Affiliations:** 1 Clinical Advanced Analytics, Global Medical Office, Fresenius Medical Care, 26020 Vaiano Cremasco, Italyfrancesco.bellocchio@freseniusmedicalcare.com (F.B.);; 2 Nephrocare Spain, Fresenius Medical Care, 08013 Barcelona, Spainabraham.rincon@freseniusmedicalcare.com (A.R.B.);; 3 Health Economics and Market Access, Fresenius Medical Care, 61352 Bad Homburg, Germany; 4 Global Digital and Innovation Technology Department, Fresenius Medical Care, 26020 Vaiano Cremasco, Italy; 5 Clinical and Therapeutic Governance EMEA, Medical Affairs, Global Medical Office, Fresenius Medical Care, 26020 Vaiano Cremasco, Italy; stefano.stuard@freseniusmedicalcare.com

**Keywords:** End Stage Renal Disease (ESRD), anemia management, erythropoiesis-stimulating agent (ESA), Artificial Intelligence (AI), personalized medicine

## Abstract

Introduction: The management of anemia in chronic kidney disease (CKD-An) presents significant challenges for nephrologists due to variable responsiveness to erythropoietin-stimulating agents (ESAs), hemoglobin (Hb) cycling, and multiple clinical factors affecting erythropoiesis. The Anemia Control Model (ACM) is a decision support system designed to personalize anemia treatment, which has shown improvements in achieving Hb targets, reducing ESA doses, and maintaining Hb stability. This study aimed to evaluate the association between ACM-guided anemia management with hospitalizations and survival in a large cohort of hemodialysis patients. Methods: This multi-center, retrospective cohort study evaluated adult hemodialysis patients within the European Fresenius Medical Care NephroCare network from 2014 to 2019. Patients treated according to ACM recommendations were compared to those from centers without ACM. Data on demographics, comorbidities, and dialysis treatment were used to compute a propensity score estimating the likelihood of receiving ACM-guided care. The primary endpoint was hospitalizations during follow-up; the secondary endpoint was survival. A 1:1 propensity score-matched design was used to minimize confounding bias. Results: A total of 20,209 eligible patients were considered (reference group: 17,101; ACM adherent group: 3108). Before matching, the mean age was 65.3 ± 14.5 years, with 59.2% men. Propensity score matching resulted in two groups of 1950 patients each. Matched ACM adherent and non-ACM patients showed negligible differences in baseline characteristics. Hospitalization rates were lower in the ACM group both before matching (71.3 vs. 82.6 per 100 person-years, *p* < 0.001) and after matching (74.3 vs. 86.7 per 100 person-years, *p* < 0.001). During follow-up, 385 patients died, showing no significant survival benefit for ACM-guided care (hazard ratio = 0.93; *p* = 0.51). Conclusions: ACM-guided anemia management was associated with a significant reduction in hospitalization risk among hemodialysis patients. These results further support the utility of ACM as a decision-support tool enhancing anemia management in clinical practice.

## 1. Introduction

Anemia of chronic kidney disease (CKD-An) is common among individuals undergoing kidney dialysis. Uncontrolled CKD-An is associated with a risk for low health-related quality of life, as well as for mortality and hospitalization [1,2]. Consequently, clinical guidelines recommend the correction of CKD-An in most dialysis patients [1,3,4,5,6,7]. The established protocol entails the administration of iron supplements and erythropoiesis-stimulating agents (ESAs) [8] to maintain hemoglobin (Hb) serum levels within the recommended target range [1,9]. Nevertheless, the effective management of renal anemia presents distinct challenges for nephrologists, with recent findings indicating that only 45–65% of hemodialysis patients consistently achieve hemoglobin concentrations within the target range [10,11,12,13].

Anemia management is a complex clinical task requiring a challenging trade-off negotiation between anemia correction and minimization of potential side effects of ESA and Iron therapy. Both hemoglobin fluctuations [14,15] and excessive ESA usage are associated with a higher risk of morbidity and mortality [16,17,18,19,20,21,22,23,24]. A meta-regression analysis found significant associations between a high ESA dose and the development of hypertension, stroke, and thrombotic events, as well as with all-cause mortality, irrespective of the achieved hemoglobin levels [25]. High doses of these compounds were likewise associated with an increased rate of arteriovenous fistula failure [26], higher risk of cardiovascular complications [25,27] and hospitalizations [28], and enhanced mortality [29,30], also among ESA hypo-responders [31]. On the other hand, hemoglobin cycling above and below the target range is associated with increased all-cause hospitalization [27,32] and cardiovascular risk [27,33], as well as higher mortality [19,33,34,35]. Hemoglobin cycling is a common condition depending on fluctuations in ESA bioavailability and bone marrow responsiveness due to transient inflammation, hydration, iron deficiency, and malnutrition [36,37].

Hence, tailoring anemia management to accommodate individual patient variances and temporal fluctuations in erythropoiesis presents a challenging yet fundamental clinical task aimed at minimizing hemoglobin fluctuations while optimizing erythropoiesis-stimulating agent (ESA) dosages. In order to provide support and standardization for medical decision-making, we have developed the Anemia Control Model (ACM) [38], an Artificial Intelligence (AI) decision support system designed to assist physicians in selecting personalized anemia therapies for their patients. Initial investigations have demonstrated that the implementation of the ACM into routine clinical practice has led to an increase in the proportion of patients achieving target hemoglobin values, a reduction in ESA dosages, and mitigation of individual hemoglobin fluctuations [39,40]. Moreover, a recent large-scale cohort study utilizing propensity-score matching has further underscored the real-world effectiveness of the ACM. This study revealed that compared with standard care approaches, ACM-guided care was associated with higher rates of hemoglobin target achievement, as well as lower incidences of severe anemia and ESA utilization for patients with hemoglobin levels above 12 g/dL [41].

In the present study, we sought to investigate the association between ACM-guided anemia care and patient-centered outcomes such as hospitalization and survival among hemodialysis patients treated in the European Fresenius Medical Care NephroCare network.

## 2. Materials and Methods

### 2.1. The Anemia Control Model (ACM)

The Anemia Control Model (ACM) is an Artificial Neural Network Algorithm that personalizes ESA and iron dosages based on the estimated individual dose-response relationship. The ACM suggests the optimal dose of ESA and iron monthly [38,39,42,43]. The ACM first simulates how future hemoglobin values would vary for different possible ESA and iron dosages, and, secondly, the ACM policy extractor assigns a utility score to each simulated ESA dosage based on a reward function anchored at pre-specified clinical targets. The simulated action achieving the highest utility score is suggested. The ACM integrates a comprehensive dataset, including current Hb levels and all Hb values within the 120 days prior to the algorithm’s execution, ferritin levels, and additional laboratory data such as albumin, calcium, C-reactive protein, leukocytes, mean corpuscular hemoglobin (MCH), mean corpuscular volume (MCV), potassium, phosphate, sodium, transferrin saturation (TSat), and overhydration status. Hemodialysis (HD) treatment data, including pre-dialysis weight, dry body weight, Kt/V, ESA and iron administration details (dose quantities, units, routes, and codes) within 140 days, are also considered. Basic patient demographics (age, sex, height, and admission date) and transfusion records within the past 120 days further contribute to the model.

The architecture of the ACM involves a cloud-based suggestion engine that processes pseudonymized patient data from hospital information systems, ensuring secure data handling. The ACM operates as a decision support tool, where recommendations are presented to nephrologists via an integrated application within the hospital’s IT environment. Nephrologists can review and either accept or reject the suggested dosages, allowing for clinical discretion and personalized patient management. This integration facilitates the seamless application of ACM’s data-driven, evidence-based recommendations into routine clinical practice, supporting the optimization of anemia management in patients with end-stage renal disease (ESRD).

The ACM was certified as a medical device within the European Community under MDR 2017/745. The legal manufacturer of ACM is Fresenius Medical Care AG (Fresenius Medical Care, Hafenstraße 9, 97424 Schweinfurt, Germany). We further corroborated ACM effectiveness in improving hemoglobin target achievement rate and reducing Erythropoietin Stimulating Agents consumption in a recent multi-center real-world evidence study [41].

### 2.2. Study Design and Participants

**Figure 1:**
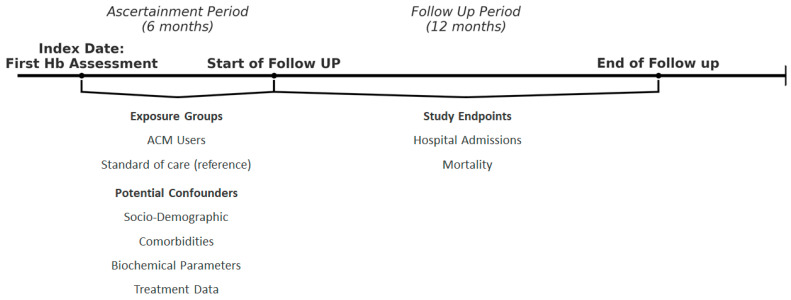
Study design diagram. This diagram illustrates the timeline and structure of the study, detailing the ascertainment period, follow-up period, exposure groups (ACM users and standard of care), potential confounders, and primary study endpoints (hospital admissions and mortality).

In this multi-center, matched, retrospective, historical cohort, observational study, we screened for eligibility all incident adult patients on chronic hemodialysis receiving care for at least 180 days in the European Fresenius Medical Care NephroCare network of Bosnia and Herzegovina, Czech Republic, France, Hungary, Italy, Poland, Portugal, Romania, Slovakia and Spain from 1 January 2014 to 31 December 2019. The study design is displayed in Figure 1. We included patients with complete information regarding biological sex, Fresenius Medical Care NephroCare admission date, renal replacement therapy onset date, and patient’s age. A Continuous Quality Improvement program called Medical Patient Review (CQI-MPR) has operated in all FMC-NephroCare clinics since 2014. The Medical Patient Review program associates extensive medical training and guidance with key performance indicator targets across the network. Under Medical Patient Review, physicians are required to test hemoglobin monthly and ferritin at least quarterly in all patients. Additionally, participating centers are required to reach pre-specified hemoglobin target achievement rates every month. Characteristics and outcomes of the Medical Patient Review program have been described elsewhere [44]. We used the first 180 days of dialysis after the date of the first hemoglobin assessment/ACM suggestion (index date) as the ascertainment period. Finally, we excluded patients with cancer and transfusions in the ascertainment period. Study endpoints were evaluated over 365 days of follow-up since the end of the ascertainment period (Figure 1).

### 2.3. Definition of Exposure Groups

#### 2.3.1. ACM Adherent Patients

Patients who were consistently treated according to ACM recommendations were allocated to the ACM adherent group. ACM does not produce any suggestions in case of errors in reporting ESA or iron dosage (i.e., wrong measurement unit of dosage, wrong route of administration, etc.), elevated frequency of missed treatment in the 4 months prior to the index hemoglobin measurement, and never for patients with a diagnosis of porphyria. Therefore, we excluded patients with less than 3 ACM suggestions during the ascertainment period. Patients in the ACM group were further classified as ACM compliant if more than 65% of suggestions were accepted by the physician and ACM non-compliant otherwise. The threshold is consistent with previous clinical research concerning the use of ACM [39,40].

#### 2.3.2. Reference Group

Patients treated in centers where ACM was not activated were included in the reference group. All patients included in this group were managed according to established clinical standards [38]. For patients in the reference group, we required that patients receive 3 or more hemoglobin assessments in the ascertainment period.

### 2.4. Covariates

We abstracted demographic (age, biological sex, ethnicity) and anthropometric information (BMI) from patients’ clinical records. Relevant comorbidities were ascertained based on the occurrence of suggestive ICD10 codes during the ascertainment period. The full list of ICD10 codes used to classify patients’ comorbidities is reported in Supplementary Table S1. All biochemical assessments occurring during the ascertainment period were averaged and abstracted from patients’ clinical charts.

### 2.5. Outcome Definition

The primary endpoint was the number of hospitalizations occurring for each patient during the study follow-up period. The secondary endpoint was the patients’ survival after the ascertainment period. We censored at the end of the follow-up period and patients leaving the FMC-NephroCare network.

### 2.6. Statistical Analysis

We computed mean and standard deviation or median and interquartile range for continuous variables as appropriate and absolute and relative frequency for categorical variables. A 1-way ANOVA, Mann–Whitney test, and χ^2^ test were used to assess differences in covariate distribution across groups as appropriate. 

#### 2.6.1. Primary Analysis

In order to simulate a randomized controlled trial and account for potential indication bias, we constructed a 1:1 matched cohort to compare ACM adherent patients with a reference group of patients without any exposure to ACM.

##### Propensity Score (PM) Estimation

Because the decision whether to accept or not the ACM monthly suggestion may depend on patients’ characteristics, we estimated a propensity score (PS) representing the likelihood that the attending physicians would consistently accept ACM suggestions for each patient treated in ACM centers included in this study. A patient was considered consistently treated in accordance with ACM if the attending physicians accepted more than 65% of software recommendations for that patient during the ascertainment period. The PM was estimated by a logistic regression model assessing the likelihood of consistent ACM acceptance (ACM adherent patient, i.e., >65% of suggestions were accepted) versus inconsistent acceptance (ACM non-adherent patient; <65% of accepted suggestions) given the full set of baseline covariates described above.

##### Matching Strategy

In order to mitigate the indication bias, we matched each ACM adherent patient to 1 corresponding patient in the reference group (i.e., patients treated in clinics where ACM was never activated). We applied an optimal matching algorithm to obtain a 1:1 matched sample. The maximum caliper allowed for matching was 0.05 and we limited to control matches with an index date and dialysis vintage difference compared to ACM cases smaller than 180 days.

##### Outcomes Estimation

We estimated the event rate ratio per 100 patients/month of hospitalization and hazard ratio of mortality using a zero-inflated negative binomial regression and Proportional Hazard regression, respectively. We accounted for the matched design of the study by adding a random intercept representing dependency within pairs.

#### 2.6.2. Secondary Analysis

Because we observed residual imbalance in Kt/V after propensity score matching, we further added Kt/V as a covariate in the model.

## 3. Results

### 3.1. Study Sample before Matching

We included 20,209 patients meeting the eligibility criteria (reference group: 17,101; ACM adherent group: 3108; Figure 2). Patients were 59.2% male (*n* = 11,962) with a mean age of 65.3 ± 14.5 years. The distribution of each variable included in the PS model by exposure group before matching is reported in Table 1.

### 3.2. Propensity Score Estimation

**Figure 3:**
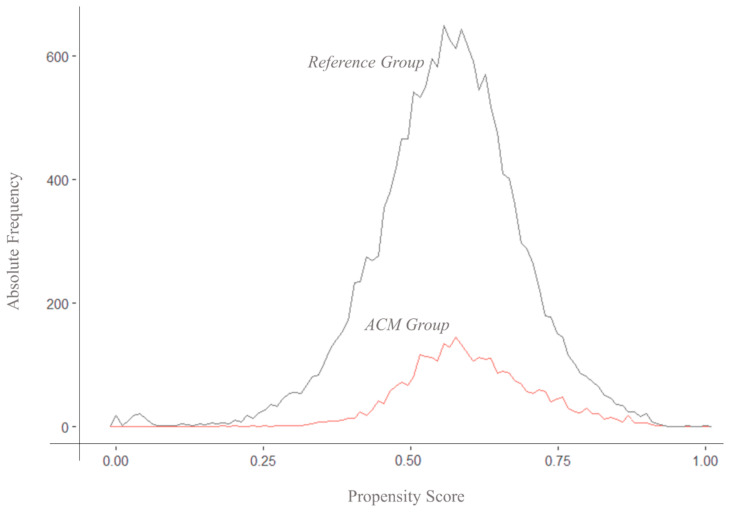
Distribution of propensity scores in the ACM and reference groups before matching. Distribution of propensity scores for the ACM group (red) and the reference group (black). The histogram demonstrates the overlap and divergence in propensity scores between the two groups, indicating the relative frequency of individuals across the range of propensity scores.

We estimated a propensity score (PS) representing the likelihood that the attending physicians would consistently accept ACM suggestions for each patient treated in ACM centers included in this study. We included in the model patients’ characteristics potentially affecting Hb target achievement based on our previous studies [45]. The distribution of propensity scores before matching is displayed in Figure 3.

The distribution of propensity scores across groups shows a wide common support region. However, a non-negligible share of ACM patients with extremely high propensity scores has only a few available matches among patients in the reference group. After matching, the propensity score in the ACM group was not significantly different compared to the reference group (ACM: 0.593 ± 0.102; reference: 0.592 ± 0.102). Among unmatched ACM patients, the propensity score was slightly higher (ACM_U_ = 0.62 ± 114).

### 3.3. Study Sample after Matching

After PS matching, both exposure groups included 1952 patients, whereas 1167 ACM adherent patients could not be matched. Unmatched ACM patients had longer dialysis vintage, were more likely to have peripheral artery disease and were more likely to have an arteriovenous fistula as vascular access. Differences in baseline characteristics between matched ACM adherent and non-ACM patients are negligible or very small in magnitude (Table 2). Of note, after matching, 19.5% (*n* = 323) in the reference group and of the ACM group (*n* = 74.6%) were treated with online hemodiafiltration (OL-HDF). Given that we observed a strong overlap between ACM activation in centers where HDF was more prevalent, we could not include this variable in the statistical model.

### 3.4. Hospitalization and Mortality Rate

The incidence of hospitalization in the whole sample before matching was 80.9/100 person/years (95% CI: 79.6–82.3/100 person/years). Hospital admission rate was lower in the ACM group compared with the reference group (ACM group: 71.3/100 person/years, 95% CI: 68.0–74.6/100 person/years; reference group: 82.6/100 person/years, 95% CI: 81.2–84.2/100 person/years; Incidence Rate Difference: 11.4, 95% CI: 7.6–15.2/100 person/years, *p* < 0.001) (Table 3).

After propensity score matching, we observed 80.9 admissions/100 person/years (95% CI: 77.8–84.1/100 person/years). Hospital admission rate was statistically lower in the ACM group compared with the reference group after matching (ACM group: 74.3/100 person/years, 95% CI: 70.2–78.7/100 person/years; reference group: 86.7/100 person/years, 95% CI: 82.4–91.6/100 person/years; Incidence Rate Difference: 12.6/100 person/years, 95% CI: 6.3–18.9/100 person/years, *p* < 0.001). During the follow-up period, 385 patients died (incidence rate: 9.89%; 95% CI: 8.93–10.91%). We observed no evidence of survival benefit for patients treated with ACM guidance during the follow-up period of 1 year (hazard ratio = 0.93; *p*-value = 0.51).

### 3.5. Secondary Analysis

Because we observed residual imbalance in Kt/V after propensity score matching, we included this variable as a confounder in a zero-inflated negative binomial model. Lower Kt/V was significantly associated with increased hospitalization risk (RR = 0.96 per each 0.1 increase in Kt/V; *p* < 0.01). The estimated risk of hospitalization in the ACM group was still 12% lower compared with the reference group (adjusted rates: ACM group, 83.6/100 person/years; reference group, 95.0/100 person/years; adjusted risk ratio: 0.88, *p* < 0.001).

## 4. Discussion

In this propensity-score-matched, real-world, historical cohort study, we observed that anemia management based on the Anemia Control Model (ACM) recommendations compared to standard of care was associated with a significant reduction in all-cause hospitalizations among hemodialysis patients. Mechanisms leading to reduced hospitalization associated with optimization of anemia management may include reduced likelihood of cardiovascular complications associated with Hb variability [27,32,33] and optimization of ESA and supplemental iron dosing [18,20,21,22,23,25,46]. While previous clinical studies have consistently shown that usage of ACM in clinical practice is associated with large ESA savings as well as improved clinical outcomes, including hemoglobin target achievement, reduced risk of severe anemia, and reduced hemoglobin cycling [39,40,41], this study further showed that improved anemia management by the use of AI-supported decision-making is associated with reduced hospitalization rates. 

ACM is a decision support system providing personalized drug dosage suggestions considering a set of commonly available clinical information such as hemoglobin and ferritin values, markers of inflammation, hydration status, and demographic variables [38]. The software is based on an artificial neural network that first simulates patient-specific dose-response relationships and then uses a reward function to select the optimal dosage, minimizing drug utilization while maximizing the likelihood of hemoglobin target achievement [38,42,43]. Usage of predictive algorithms such as ACM or other AI-assisted anemia management systems [47,48,49] may help overcome known clinical challenges, including the non-linearity of ESA dose-response relationships [49,50], the temporal discrepancy between the ESA half-life and RBCs’ lifespan (months) [51], differences in ESA responsiveness between patients [22,52], and temporal variations in bone marrow responsiveness [51,53,54].

Despite improvement in hospitalization rate, the reduction in mortality rate associated with ACM was not statistically significant. Previous studies have shown that patients exposed to higher dosages of ESA may be at higher mortality risk [28,30,55,56,57]. Conversely, studies concerning the association between hemoglobin variability, hemoglobin levels, and mortality risk obtained mixed results [27,33,52,58,59,60,61,62]. Failure to observe a statistically significant reduction in mortality rate in our study may be due to insufficient power, short follow-up time, or insufficient improvement in anemia management to translate into sizeable survival benefits.

This study has several strengths. Our large multinational sample allowed extensive adjustment for potential confounding factors and extended the generalizability of results to different populations and clinical settings. Additionally, the use of propensity score matching offers several advantages in observational studies by addressing confounding bias and approximating the conditions of a randomized controlled trial. By matching participants with similar propensity scores, it balances the distribution of observed covariates between treated and reference groups, enhancing the comparability of these groups. Despite ACM activation not depending on clinical considerations, it is a policy decision adopted at the clinic level, acceptance of ACM suggestions may be related to patients’ clinical characteristics. In our study, the distribution of propensity scores of the two groups largely overlapped, suggesting that activation and usage of ACM were poorly related to patients’ medical parameters. Nevertheless, this study is also subject to a few limitations. Observational studies cannot provide definitive proof of causality. Despite extensive adjustment by propensity score matching, residual confounding from unmeasured clinical parameters cannot be completely ruled out. Additionally, we could not match a non-trivial proportion of patients in the ACM arm. Unmatched ACM patients had longer dialysis vintage, were more likely to have peripheral artery disease, and were more likely to have an arteriovenous fistula as vascular access. The hospitalization rate among unmatched ACM patients was lower than both the reference group and matched ACM patients. Therefore, our stringent matching criteria may have led to an underestimation of ACM benefit; it is also possible, however, that the lower hospitalization rate observed among unmatched ACM patients may have been totally or partially explained by higher AVF prevalence in this group, a condition associated with lower rate of acute complication needing hospital admission [63]. Finally, we could not rule out the possibility that the unequal distribution of online hemodiafiltration (OL-HDF) treatment across exposure groups may have affected our results. In fact, there was a strong overlap between ACM activation in centers where HDF was more prevalent, a condition that prevented including this variable in the model. Even though the Convince Study, the FRENCHIE study, and the Turkish OL-HDF study did not show a statistically significant hospitalization risk reduction in the OL-HDF group [64,65,66], this benefit has been reported in previous studies [67], thus leaving the possibility that our results are confounded by an imbalance in treatment modality distribution across exposure groups. Therefore, further studies should analyze the interplay between ACM and dialysis modality in detail.

## 5. Conclusions

We observed a statistically significant association between the adoption of ACM-assisted anemia management and reduced hospitalization among hemodialysis patients. The results from this study extend on previous research showing that ACM usage improved hemoglobin target achievement and reduced the likelihood of severe anemia and hemoglobin variability while reducing ESA usage. Overall, the evidence generated in this article further supports the utility of the ACM as a decision-support tool for anemia management in clinical practice and provides the rational to assess the potential for incremental benefits of ACM among OL-HDF patients. Further studies should assess the cost-effectiveness of ACM for anemia management compared to standard of care.

## Figures and Tables

**Figure 2:**
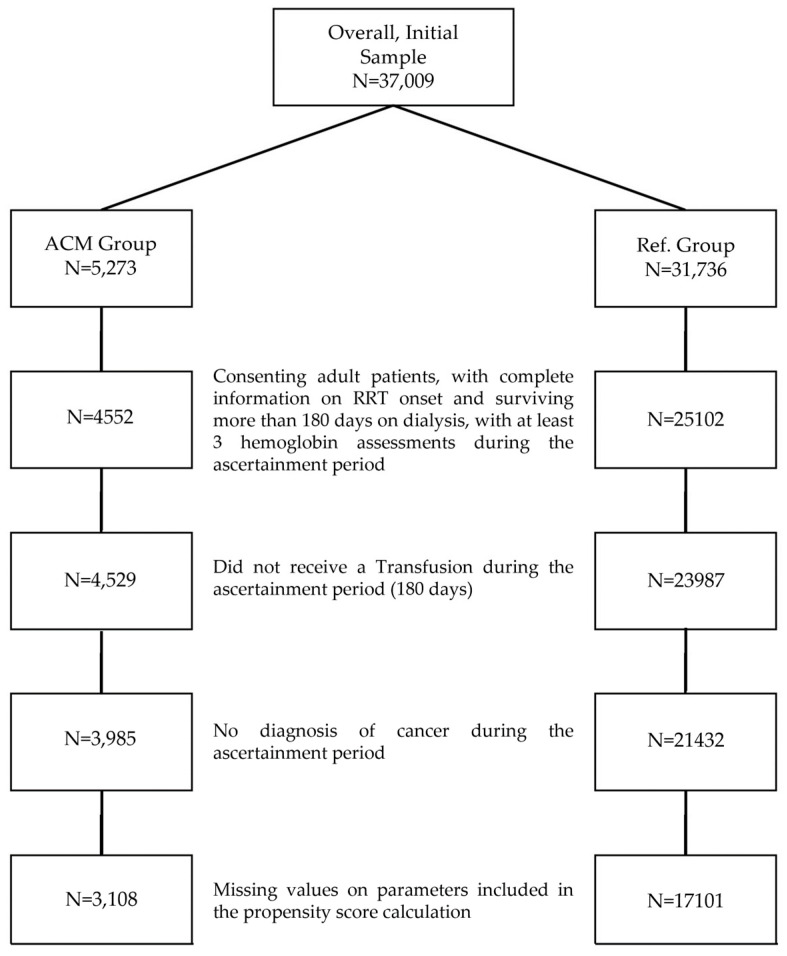
Study flowchart diagram. Flowchart depicting the selection process for the ACM and reference groups from the initial sample (N = 37,009). The ACM group (N = 5273) was refined through multiple exclusion criteria, resulting in a final sample size of N = 3108. The reference group (N = 31,736) was similarly refined to a final sample size of N = 17,101.

**Table 1:** Sample characteristics before propensity score matching.

	Exposure Groups			Significance *	
Characteristics	Whole Sample (*n* = 20,209)	ACM Group (*n* = 3108)	Reference Group (*n* = 17,101)	*p*-Value	Effect Size
	*n* (%), Mean (St.D.), or Median (IQR)				
Age	65.3 (14.5)	67.8 (14.4)	64.8 (14.4)	<0.001	0.0107
Men	11,962 (59.2)	1945 (62.6)	10,017 (58.6)	<0.001	0.0234
BMI	26.8 (6.5)	26.4 (5.4)	26.9 (6.7)	<0.001	0.0015
Dialysis vintage (years)	2.03 (4.7)	2.8 (5.26)	1.87 (4.59)	<0.001	0.2672
Vascular access				<0.001	0.1280
Catheter or graft	5578 (28.2)	592 (19.0)	4986 (29.9)		
Arteriovenous fistula	14,188 (71.8)	2517 (81.0)	11,671 (70.1)		
Missing	419 (2.1)	0 (0)	419 (2.5)		
Kt/V	1.6 (0.4)	1.9 (0.4)	1.6 (0.4)	<0.001	0.2554
Treatment time (minutes)	241.8 (13.1)	243.7 (13.1)	241.5 (13.1)	<0.001	0.0169
Hemoglobin (g/dL)	11.1 (1.2)	11.4 (1.1)	11.0 (1.2)	<0.001	0.0188
Albumin (g/dL)	3.9 (0.4)	4.0 (0.5)	3.9 (0.4)	<0.001	0.0075
Ferritin (ng/mL)	558.8 (436.5)	567.0 (349.7)	557.3 (450.8)	0.176	0.0037
Phosphate (mg/dL)	4.7 (1.4)	4.3 (1.1)	4.8 (1.4)	<0.001	0.0269
Leukocytes (10^3^/µL)	7883.2 (41,545.4)	6582.5 (1824.9)	8123.0 (45,203.6)	<0.001	0.0286
C-reactive protein (mg/L)	13.6 (22.3)	12.1 (17.0)	13.8 (23.1)	<0.001	0.0030
Transferrin saturation (%)	29.9 (12.8)	32.2 (11.9)	29.5 (12.9)	<0.001	0.0007
MCV (fL)	94.3 (6.5)	95.1 (5.9)	94.1 (6.6)	<0.001	0.0288
MCH (pg/cell)	32.9 (43.1)	32.9 (0.8)	32.9 (46.9)	0.874	0.0044
Serum sodium (mmol/L)	138.1 (4.0)	138.3 (2.6)	138.1 (4.1)	0.0042	0.0042
Serum potassium (meq/L)	4.9 (0.9)	5.0 (0.6)	4.9 (0.9)	0.0066	0.0066
Serum calcium (mg/dL)	8.8 (1.3)	9.0 (0.6)	8.8 (1.4)	0.0060	0.0059
Cerebrovascular disease	2750 (13.6)	486 (15.6)	2264 (13.2)	<0.001	0.0401
Chronic pulmonary disease	2197 (10.9)	398 (12.8)	1799 (10.5)	<0.001	0.0417
Congestive heart failure	4376 (21.7)	696 (22.4)	3680 (21.5)	0.287	0.0075
Connective tissue disorder	332 (1.6)	43 (1.4)	289 (1.7)	0.246	0.0105
Coronary artery disease	4199 (20.8)	621 (20.0)	3578 (20.9)	0.243	0.0152
Dementia	313 (1.5)	53 (1.7)	260 (1.5)	0.491	0.0115
Diabetes without complication	6155 (30,5)	214 (6.9)	871 (5.1)	<0.001	0.0052
Diabetes with organ damage	5070 (25.1)	921 (29.6)	4149 (24.3)	<0.001	0.0560
Hemiplegia	157 (0.8)	20 (0.6)	137 (0.8)	0.418	0.0157
Mild liver disease	2002 (9.9)	385 (12.4)	1617 (9.5)	<0.001	0.0419
Moderate/severe liver disease	111 (0.5)	16 (0.5)	95 (0.6)	0.880	0.0179
Peptic ulcer disease	1026 (5.1)	152 (4.9)	874 (5.1)	0.638	0.0125
Peripheral Vascular Disease	3769 (18.7)	740 (23.8)	3029 (17.7)	<0.001	0.0506

Notes: ACM = Anemia Control Model; BMI = Body Mass Index; Kt/V = dialysis dose (a measure of dialysis adequacy); MCV = mean corpuscular volume; MCH = mean corpuscular hemoglobin. The table includes mean (standard deviation) for continuous variables and count (percentage) for categorical variables. * Statistical significance was assessed using *p*-values, with effect sizes reported as Cramer’s V for categorical variables and η^2^ for continuous variables.

**Table 2:** Sample characteristics after propensity score matching.

	Exposure Groups				Significance *	
Characteristics	Whole Sample (*n* = 5071)	ACM Group (*n* = 1952)	ACM Unmatched (*n* = 1167)	Reference Group (*n* = 1952)	*p*-Value	Effect Size
	N (%) or Mean (St.D.)					
Age	66.1 (14.7)	67.6 (14.5)	68.3 (14.3)	63.3 (14.8)	<0.001	0.0413
Men	3165 (62.7)	1243 (64.0)	701 (60.1)	1221 (62.9)	0.09	0.0111
BMI	26.7 (5.6)	26.4 (5.3)	26.3 (5.5)	27.2 (6.0)	<0.001	0.0010
Dialysis vintage	2.03 (4.59)	1.25 (2.98)	5.29 (5.51)	1.05 (3.28)	<0.001	0.0004
Vascular access					<0.001	0.1410
Catheter or graft	1241 (24.8)	420 (21.6)	172 (14.7)	649 (34.2)		
Arteriovenous fistula	3766 (75.2)	1522 (78.4)	995 (85.3)	1249 (65.8)		
Missing	44 (0.9)	0 (0)	0 (0)	44 (2.3)		
Kt/V	1.8 (0.4)	1.9 (0.4)	1.9 (0.4)	1.5 (0.4)	<0.001	0.3300
Treatment time (minutes)	242.2 (13.3)	243.9 (11.8)	243.2 (15.0)	239.8 (13.2)	<0.001	0.0170
Hemoglobin (g/dL)	11.3 (1.0)	11.3 (0.9)	11.6 (1.2)	11.2 (0.9)	<0.001	0.0061
Albumin (g/dL)	3.9 (0.4)	3.9 (0.5)	4.0 (0.4)	3.9 (0.4)	<0.001	0.0000
Ferritin (ng/mL)	520.5 (371.9)	561.0 (362.0)	577.0 (328.2)	443.4 (394.3)	<0.001	0.0460
Phosphate (mg/dL)	4.4 (1.1)	4.3 (1.1)	4.2 (1.1)	4.6 (1.1)	<0.001	0.0358
Leukocytes (10^3^/µL)	6738.9 (2148.4)	6685.2 (1833.5)	6411.6 (1798.3)	6992.9 (2569.3)	<0.001	0.0094
C-reactive protein (mg/L)	11.8 (16.6)	12.4 (18.0)	11.8 (15.2)	11.3 (16.1)	0.213	0.0020
Transferrin saturation (%)	30.3 (11.8)	31.8 (11.6)	32.9 (12.3)	27.6 (11.1)	<0.001	0.0641
MCV (fL)	94.3 (6.1)	95.2 (5.9)	94.9 (5.9)	92.9 (6.3)	<0.001	0.0663
MCH (pg/cell)	32.8 (1.0)	32.9 (0.9)	32.9 (0.8)	32.8 (1.1)	<0.001	0.0049
Serum sodium (mmol/L)	138.1 (3.1)	138.2 (2.6)	138.4 (2.7)	137.9 (3.7)	<0.001	0.0044
Serum potassium (meq/L)	4.9 (0.6)	4.9 (0.6)	5.0 (0.6)	4.8 (0.6)	<0.001	0.0137
Serum calcium (mg/dL)	8.9 (0.6)	8.9 (0.6)	9.0 (0.7)	8.8 (0.6)	<0.001	0.0137
Cerebrovascular disease	736 (14.6)	296 (15.2)	192 (16.5)	248 (12.8)	0.011	0.0348
Chronic pulmonary disease	594 (11.8)	261 (13.4)	140 (12.0)	193 (9.9)	0.003	0.0535
Congestive heart failure	1117 (22.1)	425 (21.9)	275 (23.6)	417 (21.5)	0.377	0.0043
Connective tissue disorder	74 (1.5)	25 (1.3)	18 (1.5)	31 (1.6)	0.703	0.0108
Coronary artery disease	964 (19.1)	362 (18.6)	260 (22.3)	342 (17.6)	0.005	0.0127
Dementia	84 (1.7)	37 (1.9)	16 (1.4)	31 (1.6)	0.507	0.0098
Diabetes without complication	332 (6.6)	141 (7.3)	73 (6.3)	118 (6.1)	0.291	0.0226
Diabetes with organ damage	1454 (28.8)	612 (31.5)	311 (26.6)	531 (27.3)	0.003	0.0450
Hemiplegia	31 (0.6)	9 (0.5)	11 (0.9)	11 (0.6)	0.239	0.0036
Mild liver disease	549 (10.9)	204 (10.5)	179 (15.3)	166 (8.5)	<0.001	0.0324
Moderate/severe liver disease	30 (0.6)	11 (0.6)	5 (0.4)	14 (0.7)	0.578	0.0064
Peptic ulcer disease	239 (4.7)	88 (4.5)	66 (5.7)	85 (4.4)	0.232	0.0025
Peripheral vascular disease	1087 (21.5)	424 (21.8)	318 (27.2)	345 (17.8)	<0.001	0.0502

Notes: ACM = Anemia Control Model; BMI = Body Mass Index; Kt/V = dialysis dose (a measure of dialysis adequacy); MCV = mean corpuscular volume; MCH = mean corpuscular hemoglobin. The table includes mean (standard deviation) for continuous variables and count (percentage) for categorical variables. * Statistical significance refers to the comparison between ACM matched group and the reference group. It was assessed using χ^2^ or T-test where appropriate, with effect sizes reported as Cramer’s V for categorical variables and η^2^ for continuous variables.

**Table 3:** Incidence rate of hospitalization.

Group	Incidence Rate (Events/100 Person/Years)	Incidence Rate Difference (Events/100 Person/Years)	*p*-Value
*Before matching*			
Whole sample	80.9 (95% CI: 79.6–82.3)	-	-
ACM group	71.3 (95% CI: 68.0–74.6)	-	-
Reference group	82.6 (95% CI: 81.2–84.2)	11.4 (95% CI: 7.6–15.2)	<0.001
*After matching*			
Matched sample	80.9 (95% CI: 77.8–84.1)	-	-
ACM group	74.3 (95% CI: 70.2–78.7)	-	-
Reference group	86.7 (95% CI: 82.4–91.6)	12.6 (95% CI: 6.3–18.9)	<0.001

Comparison of hospitalization incidence rates and rate differences between ACM and reference groups before and after matching with 95% confidence intervals and *p*-values based on zero-inflated negative binomial regression.

## Data Availability

The data that support the findings of this study are not publicly available due to privacy reasons, but they can be obtained from the corresponding author, Luca Neri, upon reasonable request.

## Supplementary Materials

The following supporting information can be downloaded at: https://www.mdpi.com/article/10.3390/biomedicines12102219/s1, Table S1: Operative definition of comorbidity classes based on ICD10 codes.
