# Supplementary material for: Usage of the Anemia Control Model Is Associated with Reduced Hospitalization Risk in Hemodialysis
Source: Biomedicines. 2024 Sep 28;12(10):2219. doi: 10.3390/biomedicines12102219 (PMC11504963; doi:10.3390/biomedicines12102219)
Supplement: Supplementary file 1 [file biomedicines-12-02219-s001.zip › biomedicines-3218074-supplementary.pdf]

Table S1. Operative definition of Comorbidity Classes based on ICD10 Codes

Comorbidity Class	ICD10 Codes
Aids	B20.5, B20.1, B22.0, B20.8, B21.7, B20.2, B20.9, B20.7, B21.2, B20.0, B22, B20, B21.9, B20.6, B20.3, B20.4, B21.3, B21.1, B22.7, B21.8, B21.0, B24, B20-B24, B22.1, B21, B22.2
Cerebrovascular Disease	I60.6, I66.0, I67.3, G46.2*, G46.5, I61.6, I67.80, I66.9, I62.9, I62.1, I62, I67.9, I62.02, I61.8, I67.11, G46.5*, G45.4, G46.7, I69.8, I66.4, G46.8, I68.2, I60, I67.7, G45.9, I63.0, I63.4, I61, I65, I60-I69, I67.10, I60.4, I66.2, I68.0, G46.2, I63, I60.1, I65.8, I67, I61.1, G46.4, I68, I65.2, G45.2, I67.0, G46.1, I67.88, I60.7, G45.3, I68.2*, I69.3, I65.9, I61.9, G46.0, I65.1, I62.00, I68.1, I69.0, I60.9, I67.2, G45.0, G45.8, I65.0, I61.4, I68.1*, I69, I63.8, G46.7*, G46.3*, I63.5, I68*, G46.4*, I65.3, I60.8, I67.5, I63.9, I61.3, G46, I66, H34.0, G46.8*, G46.0*, I63.1, G46.6, I67.6, I69.4, I66.3, G46.3, I63.6, I61.0, I60.2, I61.5, G46.6*, G45.1, I64, I62.09, I61.2, I60.3, I69.2, I60.5, G46.1*, I63.2, I68.8*, I68.0*, G46*, I66.8, I62.0, I63.3, I69.1, I67.8, G45, I62.01, I66.1, I67.4, I60.0, I67.1, I68.8
Chronic Pulmonary Disease	J67, J70.1, I27.8, J45.8, J44.1, J44.9, J63.5, J45.1, J70.3, J66.0, J40-J47, J63.3, J67.7, J43.2, J66.1, J67.9, J67.6, J63.2, J45, J44.0, J62.0, J46, J43.9, J41.0, J41.8, J43, J67.4, J66, J67.1, J60-J70, J66.8, J47, J67.8, J67.0, J65, J63.4, J44, J63.1, J40, J67.5, J41.1, J45.9, J66.2, J43.0, J67.2, J44.8, J42, J62.8, J64, J45.0, J63.0, J61, I27.9, J63, J43.8, J41, J60, J43.1, J67.3, J63.8, J62, J68.4
Congestive Heart Failure	I42.7, I50.0, P29.0, I11.0, I43.1*, I42.8, I50.19, I50.00, I42.5, I50.12, I43.0*, I50.04, I43.8*, I42.80, I50.14, I43.0, I50.02, I42.9, I13.0, I50.9, I42.88, I50, I42.7#, I50.03, I50.13, I43, I09.9, I50.05, I42.0, I43*, I43.8, I50.11, I43.2*, I43.2, I50.1, I13.2, I50.01, I42.6, I43.1

Connective Tissue Disorder	M34.9, M32.0, M34, M05.9, M05-M14, M32.0#, M05.3, M06.3, M06.0, M05.1, M06.1, M34.8, M35.3, M05.3+, M33.1, M35.1, M31.5, M32.1, M06.4, M33.0, M32, M06.2, M34.2#, M33, M05.2, M05, M06.9, M34.1, M33.2, M06, M32.8, M05.0, M32.9, M32.14, M34.2, M36.0, M05.8, M32.1+, M06.8, M34.0, M33.9
Coronary Artery Disease	I22.10, I22.01, I21.11, I21.2, I21.19, I25.3, I25.12, I24.8, I21.00, I22.83, I25.8, I21.39, I21.0, I22.80, I22.8, I21.98, I21.49, I25.9, I22.1, I21, I25.6, I21.29, I21.21, I24.0, I21.38, I24.1, I21.40, I21.41, I24, I21.1, I22.09, I22.89, I21.91, I22, I21.30, I25.16, I21.08, I25.22, I21.31, I21.13, I25.48, I21.97, I22.13, I22.08, I25.1, I21.10, I22.19, I21.43, I25.21, I22.18, I22.03, I25.4, I21.22, I21.48, I21.18, I22.0, I22.00, I25.10, I25.14, I21.90, I21.23, I22.88, I22.81, I21.33, I25.20, I21.28, I21.20, I25.15, I22.9, I21.99, I22.02, I22.11, I21.92, I21.12, I25.13, I25.19, I25, I25.11, I25.2, I21.02, I21.42, I25.29, I22.82, I21.03, I21.3, I21.4, I25.0, I25.40, I21.93, I24.9, I22.12, I21.09, I21.01, I21.32, I21.9
Dementia	F01.34, F02.31*, F01.29, F02.00*, F02.40*, F02.04*, F02.09*, F01.80, F01.0, F02.81*, G30.1, F01.31, F01.39, F02.49*, F02.42*, F02.24*, F01.10, F01.32, F00, F01.23, F01.9, F02*, F02.4, F01.14, F02.02*, G30-G32, F01.11, F00.09*, F02.43*, F01.82, F02.12*, F00-F03, F00.24*, F01.21, F00.0, F02.44*, F02.80*, F02.8*, F00.29*, F01.8, F01.84, F02.33*, F00.13*, F00.0*, F02.82*, F02.11*, F02.83*, F01.2, F00.01*, F02.2, F02.39*, F01.3, F00.21*, F02.41*, F02.1*, F02.89*, F02.23*, F00.9, F01.89, F00.22*, F02.34*, F00-F99, F01, F02.89*T58, F01.33, F00.14*, F00.03*, F02.01*, F02.10*, F00.1, F00.11*, F00.2*, F00.19*, G30.8, F02.89*G35, G30, F02.19*, F02.8, F00.1*, F02.29*, F02.3*, F02.21*, F00.9*, F02.30*, F01.20, F02.39*G20, G30.0, F01.83, F01.30, F01.12, F02.13*, F02.4*, F02.84*, F02.0*, F00.20*, F00.12*, F02.14*, F00.2, F02.0, F00.04*,

	F05.1, F01.1, F00.02*, F00*, F02.22*, F02, F02.32*, F02.89*E52, F01.13, F02.3, F03, F01.22, G30.9, F00-F09, G31.1, F00.00*, F02.2*, F02.20*, F01.81, F01.19, F00.10*, F02.1, F02.29*G10, F02.03*, F00.23*, F01.24
Diabetes Without Complication	E14.8, E14.9, E11.0, E12.8, E11.1, E13.8, E12.9, E12.1, E13.1, E14.0, E10.9, E10.1, E12.0, E10.6, E11.6, E10.8, E11.8, E13.6, E12.6, E13.0, E14.1, E14.6, E13.9, E10.0, E11.9
Diabetes With Organ Damage	E12.4, E14.2, E10.3, E13.4, E12.7, E13.3, E13.7, E10.5, E11.2, E10.7, E13.5, E12.2, E12.3, E13.2, E11.5, E10.2, E14.7, E14.3, N08.3, E12.5, E10.4, E14.4, E11.7, E14.5
Hemiplegia	G83.3, G82.0, G82.67, G82.63, G82.6, G83.9, G82.61, G04.1, G81.1, G82.66, G82, G81.0, G11.4, G83.4, G81, G81.9, G80.1, G82.60, G82.62, G80.2, G83.0, G82.3, G82.69, G82.65, G83.2, G82.4, G82.5, G82.1, G82.64, G82.2, G83.1
Metastatic Solid Tumor	C77.1, C77.9, C79.3, C79.8, C79.0, C79.4, C77.4&, C78.7&, C78, C78.31&, C80.9, C78.2, C79.39&, C79.8&, C78.2&, C79.83, C78.8, C77.3&, C79.32&, C79.2&, C77.8&, C80.0, C79.88, C77.5&, C79.2, C79.82, C78.5&, C77.5, C78.4, C79.84, C79.00, C78.5, C78.1, C79.42&, C78.8&, C80, C79.86, C78.1&, C77.4, C77.0&, C78.30&, C79.5&, C79.1&, C78.0&, C78.4&, C79.7&, C79.81, C77.1&, C79.6, C79.40&, C78.7, C78.3, C79.0&, C79, C77.2&, C78.0, C79.6&, C77.2, C79.30&, C79.1, C79.85, C77.3, C79.5, C78.39&, C79.49&, C79.41&, C78.6&, C79.7, C77, C79.9, C78.6, C77.9&, C79.31&, C77.8, C77.0
Mild Liver Disease	K76.8, K70, K74.2, K74.6, B18.19, K71.3#, B18.2, K76.3, K71.5, K70.2, B18.09, K74, B18.1, K71.7, K76.2, K74.0, K73.89, K73.2, B18.00, K71.4, K76.9, K74.3, K73.8, K74.4, K71.4#, K73, K73.80, B18.9, K70.0, B18.0, K74.1, K70.1,

	K74.5, K70.9, B18.8, K76.4, B18.10, B18, K71.3, K70-K77, K76.0, K70.3, K73.0, K73.1, K73.9, Z94.4
Moderate Or Severe Liver Disease	K72.9, I86.4, I85.9, K72.7, K72.73, K71.1, K72.72, I85.0, K72.71, I98.2, K76.5, K72.1, K72, K76.6, I85.3, K70.4, K76.7, K72.74, K72.79, I85, K72.0
Peptic Ulcer Disease	K25.0#, K25.7, K26.7#, K28.5, K27.7, K26.4, K27.3, K25.6, K26.3#, K28.0, K27.5, K26, K27.2, K28.2, K26.0, K27.4, K25.2, K28.7, K27.0, K27.1, K25.4, K26.0#, K26.3, K26.5#, K26.9#, K26.6, K26.2, K25, K26.1, K26.9, K26.6#, K26.4#, K27.6, K28.9, K25.5, K26.1#, K28, K28.6, K25.1, K25.9, K25.0, K26.7, K28.3, K26.2#, K26.5, K25.3, K27.9, K28.4, K27, K28.1
Peripheral Vascular Disease	K55.8, Z95.9, K55.1, I70.2, I71.05, I71.1, I71, I71.2, I77.1, I70.22, I79.0, I71.04, I71.5, I71.9, I70.25, I70-I79, I70.0, I71.4, K55.9, I70, I70.29, I70.1, I71.8, I71.00, I70.26, I71.03, I70.8, I71.02, I71.06, I79.2, I70.21, I73.9, Z95.8, I71.07, I70.23, I71.0, I71.01, I71.09, I73.1, I71.3, I70.9, I70.20, I70.24, I71.6, I73.8
Tumor Without Metastasis	C43.62, C43.53, C62.09&, C16.11&, C51.1, C34.37&, C76.2, C18.6, C34.02&, C40.81&, C92.0, C12&, C41.00&, C00.1&, C47.55&, C57.3, C51.12&, C96.0, C32, C72.00&, C41.04&, C50.80&, C63.0&, C08.01&, C50.40&, C53.09&, C81.2, C43.31, C47.59&, C54.0&, C34.24&, C49.39&, C16.33&, C91.9, C85, C69.4, C91.5, C34.8, C49.04&, C69.0, C49.2, C49.97&, C14.8&, C82.9, C49.13&, C94, C75.5&, C49.53&, C49.00&, C84.7, C40.19&, C51.2, C49.41&, C51.22&, C49.85&, C48.82&, C15.8&, C72.1&, C45-C49, C51.84&, C69.5, C49.23&, C51.04&, C49.32&, C41.30, C62.19&, C16.29&, C40.99&, C41.14&, C71.4, C47.87&, C17.8, C54.10&, C82.6, C54.23&, C45.7, C25.3&, C32.9&, C06.13&, C17.0, C48.19&, C76.3, C43.50, C34.07&, C08.02&, C30.1,

C57.4, C43.41, C00.4, C83.5, C15.4&, C69.21&,
C49.45&, C71.59&, C33.97&, C62.99&, C75.0,
C08.19&, C71.11&, C34.16&, C53.0, C09.9&,
C16.9, C71.02&, C40.33&, C09.1&, C71.19&,
C37, C50.0, C24.09&, C06.10&, C53.92&,
C00.1, C21.8&, C63.0, C47.80&, C71.25&,
C49.42&, C47.97&, C41.0, C51.23&, C34.80&,
C61, C08.92&, C34.17&, C71.21&, C38.4&,
C50.91&, C51.9&, C38.2, C47.26&, C41.02&,
C43.99, C49.20&, C95.7, C08.1, C18.11&,
C48.25&, C75.3&, C47.2, C71.03&, C49.99&,
C18.91&, C21.2, C18.82&, C08.03&, C43-C44,
C10.2&, C38, C34.04&, C49.11&, C75.1,
C45.2&, C11.9&, C71.92&, C73.94&, C22.7&,
C43.20, C17.3&, C24.9&, C52.93&, C81.4,
C49.82&, C16.31&, C58&, C40.29&, C54.11&,
C71.61&, C18.19&, C48.83&, C96.6, C63.2&,
C94.0, C06.14&, C18.59&, C83.2, C32.9,
C34.01&, C53.00&, C41.30&, C95.0, C45.1,
C08, C00.2, C38.1&, C03.9&, C60.0, C62.00&,
C72.5, C03.1&, C50.30&, C60.8, C49.12&,
C49.43&, C69.32&, C18.70&, C16.23&,
C48.04&, C67.5, C67.7&, C75.2, C90.1, C75.4&,
C49.47&, C48.17&, C74.9&, C83.7, C41.07&,
C04.0&, C74.1&, C31, C46.0, C93.2, C08.00&,
C71.31&, C18.29&, C16.14&, C20.91&, C17.0&,
C85.1, C50.92&, C69.63&, C49.87&, C51.24&,
C41.83&, C83.52, C10.3&, C69.90&, C16.9&,
C41.39&, C40.89&, C34.19&, C94.6, C90, C05,
C05.0&, C47.46&, C25.0, C47.99&, C21.2&,
C49.17&, C62.01&, C09.0&, C47.19&, C13.9&,
C33.92&, C83.00, C46.9, C10, C92.4, C20.92&,
C72.2, C68.1, C45, C16.02&, C26.1&, C69.91&,
C18.39&, C43.44, C74.0, C48.09&, C04.1&,
C54, C11.1, C49.80&, C11.3&, C48.18&, C65&,
C17.1&, C02.1, C63, C88.3, C10.0&, C30.0,
C40.3, C76-C80, C40.90&, C41.02, C18.80&,
C53.90&, C69.20&, C18.71&, C08.09&,
C71.10&, C06.12&, C52.99&, C00-C14, C81,
C49.6, C82.5, C21.0, C49.93&, C71.3, C18.09&,
C73-C75, C83.41, C11, C31.0, C96.9, C69.43&,

C83.9, C13.8, C54.9&, C49.50&, C10.3, C16.12&, C16.3&, C22.0, C71.33&, C43.54, C48.15&, C72.09&, C67.9&, C50.49&, C04.8, C96.1, C18.72&, C18.41&, C74.1, C51, C18.81&, C16.21&, C50.89&, C49.01&, C83.32, C53.91&, C34.87&, C22.4, C15-C26, C16.34&, C51.8&, C51.00&, C02.4&, C92.5, C69.83&, C64.80&, C11.2, C08.91&, C69.1, C34.21&, C05.9&, C41.09&, C13.2&, C62.02&, C18.3, C94.7, C47.16&, C16.5, C50.01&, C49.68&, C48.1, C83.33, C50.02&, C56.79&, C75.5, C72.2&, C85.0, C47.00&, C34.00&, C63.8, C52, C32.8, C71.69&, C67.8, C47.39&, C04.1, C17.1, C10.4, C25.2, C41.15&, C71.70&, C43.02, C49.8, C34.2, C84.5, C71.5, C41.19&, C34.84&, C83.6, C62.9, C14.2&, C48.87&, C40.9, C88.9, C49.28&, C68.9&, C50.4, C49.19&, C90.0, C48.16&, C43.33, C40.1, C41.4, C53.1, C74.0&, C48.07&, C31.1, C09.8, C24.1&, C47.6, C49.95&, C56.71&, C72.0, C40.12&, C50.20&, C72.8, C41.1, C18.52&, C43.70, C25.7, C54.1, C67.1, C53.80&, C47.10&, C00.4&, C67.3&, C25.1&, C31.8&, C43.52, C94.5, C50.12&, C06.19&, C76.3&, C56.72&, C02.2&, C72.3&, C92.3, C14.1, C69.09&, C17.2, C05.9, C00.8&, C22.0&, C26.8&, C00, C18.21&, C47.67&, C18.1, C34.05&, C00.0&, C49.38&, C84.2, C69.3, C49.03&, C69.23&, C10.9, C43.69, C85.9, C00.6, C48.88&, C94.1, C40.22&, C71.2, C15.4, C24.0, C40, C50.1, C48.22&, C06.20&, C69.10&, C15.0, C08.80&, C91.8, C41.43&, C47.37&, C45.2, C53.89&, C57.9&, C53.10&, C69.19&, C67.2, C51.9, C49.08&, C49.31&, C54.2, C71.09&, C17.3, C46.7&, C16.0, C41.40&, C71.45&, C00.5&, C71.01&, C83.0, C22.7, C70.0, C82.01, C96.4, C40.32&, C24.9, C67, C76.4&, C41.08&, C56.91&, C47.15&, C31.3, C15.9, C76, C49.62&, C05.2, C15.1, C43.13, C08.90&, C32.0&, C49.44&, C34.82&, C45.0&, C06.9, C61&, C46.3&, C48.89&, C71.72&, C69.42&, C50.22&, C18.40&, C21.8,

C25.4, C48.81&, C49.40&, C83.31, C47.86&,
C47.4, C34.86&, C71.63&, C71.23&, C71.51&,
C16.03&, C31.2&, C57.0, C92.9, C43.01,
C63.9&, C70.9, C02.8&, C41.49&, C53.02&,
C24.00&, C06.8&, C53.99&, C68, C50-C50,
C75.2&, C50.10&, C00.8, C70.1, C55, C95.2,
C41.23&, C18.01&, C74.9, C45.9&, C53.11&,
C41.10&, C39.8, C47.30&, C64, C49.14&,
C08.8, C43.10, C62.11&, C49.54&, C49.94&,
C45.1&, C71.8, C93.0, C50.41&, C43.23,
C49.63&, C34.9, C43.1, C56.92&, C49.81&,
C50.3, C71.0, C43.94, C17.2&, C52.92&, C84.8,
C88.4, C47.1, C49.49&, C47.25&, C47.56&,
C41.01&, C47.69&, C41.81&, C03.1, C31.8,
C02, C48.24&, C11.8&, C18.7, C47.29&, C30,
C71.30&, C00.3&, C65, C18.51&, C17.9, C26.1,
C54.20&, C49.59&, C71.52&, C93.9, C40.03&,
C96, C10.8, C83, C34.26&, C08.83&, C31.3&,
C70, C83.51, C47.66&, C40.30&, C51.1&,
C16.4&, C92, C47.40&, C34.31&, C76.4, C05.8,
C84.1, C69.2, C49.37&, C62.92&, C75.9,
C46.9&, C92.6, C68.1&, C40.83&, C49.64&,
C71.29&, C18.0, C02.3, C41.33&, C69, C13.9,
C49.15&, C49.55&, C00-C75, C34.36&, C18.5,
C41.12&, C49.07&, C41.21&, C75.8&, C40.00&,
C43.61, C43.29, C32.1, C49.9, C08.84&, C19&,
C41.32, C82.0, C10.1, C84.42, C57.8&, C17,
C04, C48.80&, C56.98&, C41.01, C34.09&,
C34.85&, C26, C47.89&, C49.69&, C02.11&,
C18.9, C68.0, C64-C68, C71.39&, C20.99&,
C18.30&, C69.49&, C71.66&, C76.7&, C48.00&,
C43.21, C63.7&, C49.65&, C43.74, C51.02&,
C04.8&, C51.11&, C02.0, C71.93&, C72.1,
C49.21&, C53, C02.4, C50.5, C63.9, C08.93&,
C49.34&, C76.5, C34.27&, C71.04&, C54.9,
C24, C85.7, C71.60&, C11.0&, C49.3, C57.8,
C16.30&, C43.72, C43.09, C50.90&, C63.1&,
C49.05&, C84.4, C48.8, C43.89, C18.32&,
C66&, C69.03&, C40.11&, C49.27&, C48.0,
C06.0&, C32.3, C67.5&, C47.07&, C91.2,
C60.8&, C69.30&, C40.91&, C73, C72.5&, C95,

C43.32, C76.7, C60.0&, C81.3, C47.9, C83.01,
C34.1, C71.91&, C40.82&, C49.10&, C08.11&,
C06, C90.3, C68.0&, C71.15&, C48.28&,
C69.29&, C47.65&, C43.81, C75.8, C00.2&,
C15, C49.89&, C38.1, C71.99&, C49.36&,
C41.05&, C40.0, C16.1, C31.9&, C06.29&,
C16.8, C67.4&, C43.71, C40.01&, C41.13&,
C43.4, C31.1&, C15.5, C54.13&, C14, C71.32&,
C03.0, C10.4&, C01, C49, C69.02&, C48.14&,
C57.1&, C49.84&, C22.1&, C63.2, C76.8&,
C06.21&, C83.8, C08.14&, C71.85&, C71.05&,
C50.2, C58, C40-C41, C13.1&, C67.8&, C76.0&,
C39.9&, C46.1, C83.4, C60.1, C43.84, C02.9,
C67.0&, C24.1, C94.2, C43.22, C41, C93.7,
C83.39, C08.10&, C16.4, C14.2, C51.03&,
C08.89&, C06.1, C18.31&, C38.8, C40.10&,
C54.22&, C41.22&, C34.33&, C62.10&, C05.8&,
C82.1, C83.71, C34.0, C95.1, C43.79, C16.6&,
C60.9, C41.8, C50.99&, C49.24&, C43.19,
C69.6, C49.51&, C16.2, C60.9&, C21.0&,
C22.3&, C16.13&, C47.17&, C24.01&, C45.0,
C60.1&, C38.8&, C41.06&, C71.83&, C49.4,
C82.3, C82.7, C38.0&, C49.60&, C71.43&,
C41.42&, C34.13&, C71.1, C14.0, C91.3,
C69.13&, C67.6&, C16, C50.42&, C49.02&,
C51.89&, C49.0, C08.12&, C50.51&, C67.4,
C57.7&, C18.20&, C15.3, C47.57&, C88.1,
C31.9, C34.22&, C18.8, C84.49, C33.90&,
C46.1&, C81.0, C47, C32.2&, C84.41, C22.9,
C94.8, C43.91, C49.91&, C43.51, C57.9, C96.2,
C21, C43.24, C48.03&, C57.1, C46.7, C02.10&,
C16.22&, C40.92&, C72.4, C18.99&, C50.60&,
C00.9, C22.1, C15.1&, C96.3, C47.45&,
C34.89&, C13.2, C91.0, C54.3&, C69.89&,
C50.29&, C45.9, C18.61&, C73.91&, C06.11&,
C48.2, C48.13&, C22.2, C83.3, C84.6, C54.29&,
C41.29&, C49.25&, C15.2&, C69.81&, C71.62&,
C31.0&, C49.5, C57.2, C83.70, C31.2, C49.1,
C81-C96, C51.8, C16.10&, C47.49&, C46.2&,
C68.8, C69.45&, C88.2, C49.16&, C73.99&,
C37&, C82.4, C52.90&, C50.69&, C46.8,

C43.92, C40.93&, C38.3, C33.91&, C00-C97, C16.5&, C09.1, C34.3, C24.8, C47.3, C55&, C66, C49.30&, C08.13&, C84.51, C71.80&, C47.90&, C50.52&, C71.40&, C75, C43.43, C76.1, C62, C97, C25.9&, C95.8, C84, C06.0, C71, C54.21&, C71.35&, C51.82&, C93, C75.0&, C18.69&, C43.34, C34.81&, C49.52&, C92.7, C49.83&, C51.0, C43.12, C39, C84.59, C11.2&, C22.4&, C16.3, C40.2, C50.21&, C47.09&, C81.9, C05.1, C48.26&, C51.19&, C09.9, C47.85&, C72.9, C57, C26.0&, C17.8&, C16.32&, C43.03, C71.6, C43.7, C60.2&, C43.3, C30.1&, C88, C47.27&, C45.7&, C41.9, C82.00, C67.9, C38.2&, C08.94&, C09.8&, C32.2, C51.83&, C50.39&, C83.40, C43.80, C53.81&, C51.21&, C41.2, C49.88&, C23, C34.11&, C63.8&, C00.9&, C49.22&, C41.31&, C41.80&, C43.04, C18.62&, C71.41&, C22.3, C71.81&, C50.00&, C17.9&, C96.8, C83.80, C08.81&, C32.8&, C00-D48, C34.15&, C18.22&, C72.4&, C71.49&, C56.73&, C18.2, C43.8, C50.31&, C52.91&, C49.57&, C72, C75.9&, C69.99&, C47.60&, C50.62&, C47.0, C93.3, C34.06&, C75.1&, C00&, C50.81&, C51.13&, C46, C92.1, C71.95&, C49.67&, C60.2, C38.3&, C48.20&, C02.0&, C82.2, C73.93&, C18.89&, C47.5, C48.06&, C67.3, C00.0, C25.7&, C13.0, C34.30&, C10.9&, C91.6, C06.2, C41.3, C60, C71.73&, C72.3, C15.2, C62.91&, C08.99&, C50.32&, C18.90&, C69.9, C49.98&, C21.1, C34.83&, C84.0, C25.9, C41.32&, C19, C18.49&, C50.50&, C91, C69.69&, C40.20&, C05.1&, C71.82&, C56.74&, C33.93&, C34.29&, C24.8&, C07.70&, C34.12&, C48.21&, C69.00&, C06.9&, C49.33&, C49.86&, C41.11&, C72.02&, C48.12&, C25.8&, C83.59, C41.31, C74, C48.29&, C50.11&, C69.80&, C16.8&, C43.73, C25.0&, C43.82, C34.39&, C69.39&, C14.8, C93.1, C05.2&, C71.9, C43.59, C03.0&, C41.82&, C71.12&, C00.5, C51-C58, C30-C39, C71.65&, C62.1, C03, C51.01&, C50.59&, C70.0&, C05.0, C40.21&, C20.90&,

C25, C12, C51.81&, C60-C63, C23&, C53.12&, C43.60, C43.11, C11.1&, C67.2&, C02.9&, C34, C50.8, C51.09&, C15.9&, C56, C43, C18.79&, C18, C16.20&, C38.0, C57.7, C71.50&, C53.01&, C18.00&, C69.60&, C22.9&, C88.7, C92.8, C73.92&, C72.8&, C81.0&, C71.5&, C84.52, C43.00, C16.19&, C68.9, C48.10&, C48.23&, C00.3, C49.48&, C85.2, C51.29&, C50.6, C33.96&, C50.93&, C04.9&, C11.9, C54.8&, C02.3&, C15.3&, C49.35&, C25.2&, C47.06&, C10.8&, C71.90&, C43.40, C38.4, C69.59&, C48.27&, C90.2, C75.3, C69.82&, C67.6, C50, C40.23&, C43.93, C34.20&, C84.3, C48.85&, C47.8, C49.26&, C43.39, C71.89&, C91.1, C43.0, C69.8, C47.20&, C83.35, C71.7, C48.05&, C09, C62.90&, C95.9, C16.24&, C25.8, C10.2, C14.0&, C84.43, C72.01&, C43.5, C63.7, C26.8, C33, C20, C40.02&, C83.1, C53.19&, C54.14&, C15.0&, C43.90, C34.03&, C54.3, C76.1&, C26.0, C34.34&, C67.7, C41.89&, C83.89, C48.02&, C48.86&, C49.18&, C47.47&, C50.09&, C67.1&, C43.63, C32.1&, C64.88&, C39.0, C43.9, C04.0, C10.1&, C02.2, C69.92&, C43.14, C08.0, C71.20&, C82, C49.90&, C94.3, C46.2, C96.5, C14.1&, C43.49, C76.5&, C46.8&, C62.0, C18.10&, C53.8, C49.09&, C75.4, C40.8, C47.96&, C03.9, C68.8&, C25.1, C34.25&, C81.7, C00.6&, C08.82&, C46.0&, C22.2&, C41.41&, C18.50&, C57.2&, C72.9&, C88.0, C71.42&, C16.39&, C26.9, C69.53&, C18.60&, C33.94&, C76.8, C30.0&, C39.9, C41.20&, C92.2, C51.14&, C13.1, C34.35&, C43.6, C54.0, C18.4, C04.9, C76.0, C91.7, C49.06&, C57.3&, C56.00&, C54.8, C16.6, C43.42, C32.0, C25.4&, C07.71&, C08.9, C67.0, C50.19&, C50.82&, C16.00&, C84.9, C73.90&, C54.19&, C16.04&, C07, C10.0, C71.74&, C43.2, C69.93&, C53.9, C01&, C69.40&, C94.4, C18.02&, C15.5&, C18.42&, C47.35&, C62.12&, C09.0, C46.3, C11.3, C76.2&, C43.64, C54.12&, C33.95&, C47.36&,

C34.10&, C97-C97, C40.80&, C57.4&, C40.39&,
C50.61&, C49.96&, C13.8&, C83.34, C70.9&,
C48.84&, C49.56&, C71.79&, C71.22&, C57.0&,
C40.13&, C34.32&, C13, C11.8, C34.14&,
C49.29&, C06.15&, C40.31&, C70.1&, C48,
C13.0&, C39.8&, C25.3, C18.12&, C33.99&,
C22, C71.13&, C02.8, C11.0, C81.1, C51.2&,
C16.09&, C34.23&, C39.0&, C96.7, C69.50&,
C50.9, C71.71&, C71.00&, C16.01&, C15.8,
C49.61&, C47.50&, C24.02&, C91.4, C69-C72,
C43.83, C43.30, C63.1, C32.3&, C06.8,
C41.03&, C21.1&
